# Screening of Phenolic Compounds in Australian Grown Berries by LC-ESI-QTOF-MS/MS and Determination of Their Antioxidant Potential

**DOI:** 10.3390/antiox10010026

**Published:** 2020-12-29

**Authors:** Vigasini Subbiah, Biming Zhong, Malik A. Nawaz, Colin J. Barrow, Frank R. Dunshea, Hafiz A. R. Suleria

**Affiliations:** 1 Faculty of Veterinary and Agricultural Sciences, School of Agriculture and Food, The University of Melbourne, Parkville, VIC 3010, Australia; vsubbiah@student.unimelb.edu.au (V.S.); bimingz@student.unimelb.edu.au (B.Z.); fdunshea@unimelb.edu.au (F.R.D.); 2 Commonwealth Scientific and Industrial Research Organisation (CSIRO), Agriculture and Food, 671 Sneydes Road, Private Bag 16, Werribee, VIC 3030, Australia; malik.nawaz@csiro.au; 3 Centre for Chemistry and Biotechnology, School of Life and Environmental Sciences, Deakin University, Waurn Ponds, VIC 3217, Australia; colin.barrow@deakin.edu.au; 4 Faculty of Biological Sciences, The University of Leeds, Leeds LS2 9JT, UK

**Keywords:** fruit berries, blackberries, blueberries, red raspberries, strawberries, polyphenols, antioxidant activity, HPLC-PDA, LC-MS/MS

## Abstract

Berries are grown worldwide with the most consumed berries being blackberries (*Rubus* spp.), blueberries (*Vaccinium corymbosum*), red raspberries (*Rubus idaeus*) and strawberries (*Fragaria* spp.). Berries are either consumed fresh, frozen, or processed into wines, juices, and jams. In recent times, researchers have focused their attention on berries due to their abundance in phenolic compounds. The current study aimed to evaluate the phenolic content and their antioxidant potential followed by characterization and quantification using LC-ESI-QTOF-MS/MS and HPLC-PDA. Blueberries were highest in TPC (2.93 ± 0.07 mg GAE/g_f.w._) and TFC (70.31 ± 1.21 µg QE/g_f.w._), whereas the blackberries had the highest content in TTC (11.32 ± 0.13 mg CE/g_f.w._). Blueberries had the highest radical scavenging capacities for the DPPH (1.69 ± 0.09 mg AAE/g_f.w._), FRAP (367.43 ± 3.09 µg AAE/g_f.w._), TAC (1.47 ± 0.20 mg AAE/g_f.w._) and ABTS was highest in strawberries (3.67 ± 0.14 mg AAE/g_f.w._). LC-ESI-QTOF-MS/MS study identified a total of 65 compounds including 42 compounds in strawberries, 30 compounds in raspberries, 28 compounds in blueberries and 21 compounds in blackberries. The HPLC-PDA quantification observed phenolic acid (*p*-hydroxybenzoic) and flavonoid (quercetin-3-rhamnoside) higher in blueberries compared to other berries. Our study showed the presence of phenolic acids and provides information to be utilized as an ingredient in food, pharmaceutical and nutraceutical industries.

## 1. Introduction

Berries are widely grown and consumed in Europe, America and Australia [1]. They are either consumed fresh, frozen or processed into wines, juices and jams [2]. In 2018, 116, 585 tonnes of fresh berries were produced in Australia and worth $911.4 million [3]. Blackberry (*Rubus* spp.), blueberry (*Vaccinium corymbosum*), red raspberry (*Rubus idaeus*) and strawberry (*Fragaria* spp.) are most commonly eaten berries [4]. In recent times, berry fruits have garnered the interest of the researchers around the world due to their high content and wide range of positive health promoting phenolic compounds [5].

Phenolic compounds are often known as phytonutrients, secondary metabolites or dietary bioactive compounds [6]. They have one or more aromatic ring and at least two hydroxyl groups [7]. Classification of phenolic compounds are based on their source of origin, biological function and chemical structure [8]. Phenolic compounds are divided into classes such as flavonoids, tannin, stilbenes, lignans [9], coumarins [10] and phenolic acids [6]. Majority of phenolic compounds are synthesized from the phenylpropanoid pathway [8].

The most abundant phenolic compounds present in berries are anthocyanidins, proanthocyanidins, kaempferol, quercetin, myricetin, *p*-Coumaric acid, caffeic acid, ferulic acid, *p*-hydroxybenzoic acid, gallic acid, ellagic acid, ellagitannin, flavonols, phenolic acids, and flavan-3-ols [11]. Anthocyanidins considerably contribute colour to berries such as dark red, blue green or purple, which attracts the consumers [2]. Phenolic compounds including flavonoid and phenolic acid concentrations differ due to the climate, varieties and the harvest time [2,4].

Phenolic compounds present in different berries can prevent excessive free radicals and have positive health benefits such as anti-carcinogenic, anti-inflammatory activities [11], anti-bacterial, anti-diabetics [1], prevent neurodegenerative diseases such as Alzheimer’s disease, Parkinson’s disease, prion disease, and motor neurone disease [12,13], decrease the level of blood pressure, improvement of plasma lipid profile and endothelial function [6]. In blueberries, chlorogenic acid plays a major role in antioxidant activity. Raspberries and blackberries are rich in cyanidin glycosides having high antioxidant activity whereas, strawberries have higher content of pelargonidin-3-glucoside, which are relatively weak antioxidants [5].

Phenolic compounds can be extracted using various organic solvents and evaluated using various in vitro spectrophotometric-based assays [14]. Different solvents can be used in extraction of phenolic compounds such as water, ethanol, methanol, acetone and hexane or their combinations [15]. The phenolic content can be assessed using various assays such as total phenolic content (TPC), total flavonoid content (TFC) and total tannin content (TTC). Different types of in vitro methods such as 2,2′-diphenyl-1-picrylhydrazyl (DPPH) antioxidant assay, the 2,2′-azino-bis-3-ethylbenzothiazoline-6-sulfonic acid (ABTS) and the ferric reducing-antioxidant power (FRAP) assay can be used to assess the antioxidant activity [16].

Identification, quantification and characterisation of phenolics extracted from berries can be achieved by different developed analytical methodologies [14]. Liquid chromatography integrated with electrospray-ionization, triple quadrupole and two mass spectrometry (LC-ESI-QTOF-MS/MS) is a highly sensitive tool used to identify various phenolic compounds, while high-pressure liquid chromatography (HPLC) combined with photodiode array detector (PDA) is mostly used for quantification purposes of bioactive compounds [17]. Previously, HPLC and LC-MS analysis of strawberries and blueberries showed the presence of some phenolic compounds including *p*-coumaric acid derivatives, pelargonidin, quercetin, myricetin, kaempferol and cyanidin [18] whereas, raspberries and blackberries are rich sources of ellagic acid [19].

While various studies have characterised the phenolic compounds in the berries, there are limited studies available on characterisation of phenolic compounds from Australian grown berries. For example, genetic diversity and the environmental factors may have induced some diversity in phenolic compounds of native Australian grown berries. Therefore, in the current study, we extracted phenolic compounds from Australian grown blackberries, blueberries, raspberries, and strawberries, and analysed for their antioxidant potential. Further, the identification, characterization and quantification of phenolic compounds were obtained through LC-ESI-QTOF-MS/MS and HPLC photodiode array (PDA). This outcome of the current study will provide sufficient information on the phenolic content and antioxidant properties of the native Australian grown berries to promote their usage in the food and pharmaceutical industries.

## 2. Materials and Methods

### 2.1. Chemicals and Reagents

The chemicals used in the extraction and characterisation of phenolic compounds were of analytical grade. Standards for antioxidant assays including gallic acid, quercetin, catechin and L-ascorbic acid were purchased from Sigma-Aldrich (St. Louis, MO, USA). Chemicals for antioxidant assays including Folin-Ciocalteu’s phenol reagent, aluminium chloride hexahydrate, vanillin, 2,2′-diphenyl-1-picrylhydrazl (DPPH), ferric (III) chloride anhydrous, 2,4,6-tripyridyl-s-triazine (TPTZ), potassium persulfate, 2-2′-azino-bis(3-ethylbenz-thiazoline-6-sulphonate) (ABTS), 3-ethylbenzothiazoline-6-sulphonic acid were purchased from Sigma-Aldrich (St. Louis, MO, USA). Anhydrous sodium carbonate and sodium acetate (hydrated) were purchased from Chem-Supply Pvt Ltd. and Ajax Finecham, respectively (VIC, Melbourne, Australia). Anhydrous sodium acetate, hydrochloric acid, ethanol, glacial acetic acid, and acetic acid were purchased from Thermo Fisher Scientific Inc (Waltham, MA, USA). 98% sulphuric acid was procured from RCI Labscan Ltd. (Bangkok, Thailand). HPLC grade methanol, acetic acid and acetonitrile were purchased from Fisher chemical company (San Jose, CA, USA). The standards used in HPLC including protocatechuic acid, chlorogenic acid, quercetin, quercetin-3-*O*-glucoside, quercetin-3-galactoside, quercetin-3-glucuronide, quercetin-3-rhamnoside, kaempferol, kaempferol-3-*O*-glucoside, caffeic acid, *p*-hydroxybenzoic acid, syringic acid, sinapic acid, gallic acid, caftaric acid, catechin, epicatechin, epicatechin gallate, ferulic acid and *p*-coumaric acid were purchased from Sigma Aldrich (St. Louis, MO, USA). Milli-Q water (deionized), by Millipore Milli-Q Gradient Water Purification System (Darmstadt, Germany).

### 2.2. Sample Preparation

Fruit berries (blueberries, blackberries, strawberries, and raspberries) required for this study were produced in different regions of Victoria. Freshly ripened berries were harvested, distributed and marketed within (1–3 days) and procured from a local market in Melbourne, Victoria, Australia. The berries were washed and blended into a slurry by using a 1.5 L electric blender (Russell Hobbs Classic, model DZ-1613, Melbourne, VIC, Australia). The slurry samples were stored in −20 °C for further analysis.

### 2.3. Extraction of Phenolic Compounds

The phenolic compounds present in the slurry 5 g were extracted with 20 mL 70% ethanol by modifying our previously published protocol of Gu, et al. [20]. Extracts of the berries were then prepared by homogenising the slurry samples in Ultra-Turrax T25 Homogenizer (IKA, Staufen, Germany) for 30 s at 10,000 rpm. Homogenised samples were incubated in a shaking incubator (ZWYR-240 incubator shaker, Labwit, Ashwood, VIC, Australia) for 12 h at 4 °C for 120 rpm. Subsequently after incubation, the samples were centrifuged by Hettich Refrigerated Centrifuge (ROTINA380R, Tuttlingen, Baden-Württemberg, Germany) at 5000 rpm for 15 min at 4 °C. For LC-ESI-QTOF-MS/MS and HPLC-PDA, the extract was filtered using a syringe filter (Thermo Fisher Scientific Inc., Waltham, MA, USA) of size 0.45 µm.

### 2.4. Estimation of Phenolic Compounds and Antioxidant Assay

For phenolic estimation (TPC, TFC and TTC) and for total antioxidant capacity determination (DPPH, FRAP, ABTS and TAC) the analysis were performed according to our previously published methods in Tang, et al. [21]. Absorption data was attained using a Multiskan^®^ Go microplate photometer (Thermo Fisher Scientific Inc., Waltham, MA, USA).

#### 2.4.1. Determination of Total Phenolic Content (TPC)

The TPC content in the berries was quantified by using Folin-Ciocalteu’s method as described in Samsonowicz, et al. [22] with some modifications. 25 µL extract, 25 µL Folin-Ciocalteu’s reagent solution (1:3 diluted with water) and 200 µL water were added into the 96-well plate (Costar, Corning, NY, USA). The reaction mixture was then incubated for 5 min in the dark at room temperature (~25 °C). To the reaction mixture, 25 µL of 10% (*w*:*w*) sodium carbonate was added and incubated for 60 min at 25 °C. Absorbance was measured at 765 nm using spectrophotometer (Thermo Fisher Scientific, Waltham, MA, USA). Gallic acid standard with concentration ranging from 0 to 200 µg/mL was used to prepare the standard curve and the TPC content was expressed in mg of gallic acid equivalents per gram on the basis of fresh weight (f.w.) (mg GAE/g of sample).

#### 2.4.2. Determination of Total Flavonoid Content (TFC)

The TFC was quantified by using aluminium chloride method described in Stavrou, et al. [23] with few modifications. 80 µL extract, 80 µL of 2% aluminium chloride and 120 µl of 50 g/L sodium acetate solution were added into the 96-well plate. The reaction mixture was incubated in dark room for 2.5 h. Absorbance was measured at 440 nm. Quercetin calibration curve with concentration (0–50 µg/mL) was used to determine TFC and expressed in mg quercetin equivalents per gram of sample (mg QE/g_f.w._).

#### 2.4.3. Determination of Total Tannin Content (TTC)

The vanillin sulphuric acid method was used to determine the total tannin content present in the extract with some modifications according to Haile and Kang [24]. 25 µL of 32% sulphuric acid, 25 µL of sample extract and 150 µL of 4% vanillin solution were added to 96-well plate and incubated for 15 min in the dark room. The absorbance was measured at 500 nm. Catechin calibration curve with concentration from 0 to 1 mg/mL used for estimation of TTC and expressed in mg catechin equivalents (CE) per g of sample weight (mg CE/g_f.w._).

#### 2.4.4. 2,2′-Diphenyl-1-picrylhydrazyl (DPPH) Assay

The DPPH method was used for estimation of free-radical scavenging activity of the berries by modifying the method of Ouyang, et al. [25]. DPPH (4 mg) was dissolved in 100 mL of analytical grade methanol for DPPH radical solution. 40 µL of extract and 260 µL of DPPH solution added to 96-well plate and were vigorously shaken in the dark for 30 min at 25° C. The absorbance was measured at 517 nm. Ascorbic acid standard curve with concentration ranging from 0 to 50 µg/mL was used to determine the DPPH radical scavenging activity and expressed in mg of ascorbic acid equivalent per gram (mg AAE/g_f.w._) of sample.

#### 2.4.5. Ferric Reducing Antioxidant Power (FRAP) Assay

In the FRAP assay, at low pH oxidised Fe^3+^ colourless is reduced into a blue colour Fe^2+^ tripyridyltriazine (TPTZ) by the action of electron-donating antioxidants [26]. This assay has been used to estimate the antioxidant capacity in berries with some modification of Sogi, et al. [27]. At the ratio 10:1:1, 300 mM sodium acetate solution, 10 mM TPTZ solution and 20 mM Fe [III] solution was mixed to prepare the FRAP solution. 20 µL of the extract and 280 µL prepared dye solution was added to a 96-well plate and incubated for 10 min at 37 °C. The absorbance was measured at 593 nm. Ascorbic acid standard curve with concentration ranging from 0–150 µg/mL was used to determine the FRAP values and expressed in mg of ascorbic acid equivalent per gram of sample (mg AAE/g_f.w._).

#### 2.4.6. 2,2′-Azino-bis-3-ethylbenzothiazoline-6-sulfonic acid (ABTS) Assay

ABTS radical cation decolourization assay was used to determine the free radical scavenging activity of samples with few modifications as described in Rajurkar and Hande [26]. The ABTS^+^ stock solution was prepared by addition of 5 mL of 7 mM ABTS solution and 88 µL of 140 mM potassium persulfate, the reaction mixture incubated in the dark room for 16 h. 10 µL of the extract and 290 µL dye solution was added to the 96-well plate and incubated for 6 min at 25 °C. The absorbance was measured at 734 nm. The antioxidant potential was calculated using the standard curve of ascorbic acid with concentration ranging from 0 to 150 µg/mL and was expressed in ascorbic acid equivalents (AAE) in mg per gram of sample.

#### 2.4.7. Total Antioxidant Capacity (TAC)

The phosphomolybdate method was used to estimate the total antioxidant capacity as described in Prieto, et al. [28]. Sulphuric acid (0.6 M), 0.028 M sodium phosphate and 0.004 M ammonium molybdate were mixed to form phosphomolybdate reagent. 40 µL extract and 260 µL of phosphomolybdate reagent added to the 96-well plate and incubated at 95 °C for 10 min. The absorbance was measured at 695 nm upon the reaction mixture, cooling down to the room temperature. TAC was determined by using the ascorbic acid standard curve with concentration of 0–200 μg/mL and expressed in mg ascorbic acid equivalents (AAE) per g of fresh sample weight.

### 2.5. Characterization of Phenolic Compounds by LC-ESI-QTOF-MS/MS Analysis

Extensive characterisation of phenolic compounds of four different berries were carried out using the LC-ESI-QTOF-MS/MS and method followed as described by Suleria, Barrow and Dunshea [17]. An Agilent 1200 series of HPLC (Agilent Technologies, Santa Clara, CA, USA) connected via electrospray ionisation source (ESI) to the Agilent 6530 Accurate-Mass Quadrupole Time-of-Flight (Q-TOF) LC/MS (Agilent Technologies, Santa Clara, CA, USA). The separation was carried out using a Synergi Hydro-Reverse Phase 80 °A, LC column 250 × 4.6 mm, 4 μm (Phenomenex, Torrance, CA, USA) with temperature 25 °C and sample temperature at 10 °C. HPLC buffers were sonicated using 5 L Digital Ultrasonic water bath (Power sonic 505, Gyeonggi-do, Korea) for 10 min at 25 °C. The sample injected was 6 µL and the flow rate was set at 0.8 mL/min. The system utilizes a binary solvent delivery as follows: Mobile phase A: 98% water and 2% Acetic acid; Mobile phase B: acetonitrile, water and acetic acid solution (50:49.5:0.5). The condition set for the program was carried out as following: 0 min with 10% B, 20 min with 25% B, 30 min with 35% B, 40 min with 40% B, 70 min with 55% B, 75 min with 80% B, 77 min with 100% B, 79 min with 100% B, 82–85 min with isocratic 10% B. Both positive and negative modes were applied for peak identification. Nitrogen gas has been used as nebulizer and drying gas at 45 psi, with flow rate of 5 L/min at 300 °C. Capillary and nozzle voltage was placed at 3.5 kV and 500 V respectively and the mass spectra were obtained in the range of 50–1300 amu. Further, MS/MS analyses were carried out in automatic mode with collision energy (10, 15 and 30 eV) for fragmentation. Data acquisition and analysis were performed using Agilent LC-ESI-QTOF-MS/MS Mass Hunter workstation software (Qualitative Analysis, version B.03.01, Agilent).

### 2.6. HPLC–PDA Analysis

The quantification of phenolic compounds present in the berries were executed by Agilent 1200 series HPLC (Agilent Technologies, CA, USA) coupled with a photodiode array detector (PDA) as described by Feng, et al. [29]. The sample injected was 20 µL and the wavelengths used for detection of the samples were 280 nm, 320 nm, 370 nm. Column and conditions were the same as described in LC-ESI-QTOF-MS/MS analysis. Standard calibration curves were used to detect the compounds found in sample. Data acquisition and analysis were performed using Agilent LC-ESI-QTOF-MS/MS Mass Hunter workstation software (Qualitative Analysis, version B.03.01, Agilent).

### 2.7. Statistical Analysis

The data of the phenolic content and the antioxidant assays is represented as the means ± standard deviation and one-way analysis of variance (ANOVA) was used to test for differences in mean values between different samples, followed by Tukey’s honestly significant differences (HSD) multiple rank test at *p* < 0.05. ANOVA was performed by Minitab Program for Windows version 18.0 (Minitab, LLC, State College, PA, USA).

## 3. Results and Discussion

### 3.1. Phenolic Compound Estimation (TPC, TFC and TTC)

Berries are rich source of phenolic compounds [5] and in our study, different berry extracts were analysed for estimation of phenolic compounds including TPC, TFC and TTC (Table 1).

Folin-Ciocalteu’s reagent method allows the estimation of all the phenolic compounds present including the flavonoids, anthocyanin and non-flavonoid phenolic compounds and are expressed in gallic acid equivalent (GAE/g_f.w._). In this study, the highest concentrations of total phenolic compounds were present in blueberries with 2.93 ± 0.07 mg GAE/g and the lowest concentration was observed in raspberries with 1.52 ± 0.12 mg GAE/g. In previous studies, TPC value of the methanolic extract of blueberries and blackberries ranged between 424.84–819.12 mg GAE/100 g [30] and 192.8–329.1 mg/100 g [31], respectively, while the TPC found in raspberries was 1776.02–1137.25 mg GAE/kg [32] and strawberries was 225 mg/100 g [33]. Previously, Abdelrahman, et al. [34] also reported higher concentration in these berries as compared to our study. The TPC values reported in the literature were found to be similar to the values recorded in this study (Table 1). This difference in the total phenolic compounds in the samples might be due to environmental factors, such as light, temperature, agronomic practices and genetic variation of the berries [31].

Flavonoids have gained attention due to their antioxidant activity and are an important index for nutritional assessment in food ingredients [35]. The TFC was determined by the aluminium chloride method and the TFC in this study ranged between 70.31 ± 1.21 µg QE/g and 14.31 ± 0.13 µg QE/g. The highest TFC was observed in blueberries with 70.31 ± 1.21 µg QE/g and the lowest in strawberries with 14.31 ± 0.13 µg QE/g. In previous studies, the TFC value were 30.44–91.69 mg QE/100 mg in blueberries [36], blackberries leaves with 449.00–715.00 mg QE/L based on their extraction temperature ranging between 40–80 °C [37], strawberries with 14.6 ± 3.0 mg QE/100 g [38] and raspberries 73.70–51.14 mg QE/100 g_f.w._ [39] which showed almost similar values to our study.

The TTC in our selected berries ranged between 11.32 ± 0.13 and 0.97 ± 0.13 mg CE/g. Blackberries had the highest tannin content (11.32 ± 0.13 mg CE/g_f.w._) followed by blueberries (7.41 ± 0.09 mg CE/g), strawberries (2.37 ± 0.09 mg CE/g) and raspberries (0.97 ± 0.13 mg CE/g). Previously, few studies have been conducted to calculate the total tannin content in different berries. According to Heinonen [40], red raspberries and strawberries are very rich in tannin. The concentration of tannin present in the methanolic extracts of blueberries, raspberries, blackberries were 160, 120 and 80 mg/100 g [41]. Blueberries extracted with 70% acetone and 95% ethanol had higher concentration of tannin, when compared to our study [42]. The tannin recorded in our study were lower than the literature which might be due to environmental factors, such as light, temperature, agronomic practices and genetic variation of the berries [31].

### 3.2. Antioxidant Activity (DPPH, FRAP, ABTS and TAC)

Antioxidant activity is the ability of redox molecules to scavenge free radicals present in the food and biological systems [30]. The antioxidant capacity of the four different berries were determined by DPPH, FRAP, ABTS and TAC assays and expressed in ascorbic acid per gram (AAE/g_f.w._) of sample as mentioned in Table 1.

In DPPH assay, the DPPH radical is reduced in the presence of the hydrogen and the electron donating antioxidants. Similarly, antioxidants derived from plants can reduce free radicals in food [43]. The antioxidant potential concentration varied between 1.69 ± 0.09 to 1.11 ± 0.12 mg AAE/g. Blueberries had the highest DPPH free radical scavenging activity with 1.69 ± 0.09 mg AAE/g_._ followed by raspberries (1.41 ± 0.11 mg AAE/g), blackberries (1.12 ± 0.07 mg AAE/g) and strawberries (1.11 ± 0.12 mg AAE/g). In the previous study, the free radical scavenging activity of blueberries were observed to be 65.07 ± 0.04 mg AAE/g [44], raspberries were 395.80 AAE/g [44], strawberries ranged between 3.33–21.08 mg AAE/g_d.w._ [45] and blackberries leaves with 111.5 mg AAE/g_d.w._ [46] which showed higher values when compared to our study. The difference in results might be due difference in varieties, growing region, extraction solvent, solute to solvent ratio, harvesting season and maturation stages of berries.

The FRAP assay was also conducted to measure the antioxidant capacity of the berries. In this assay, the electron transfer method was used to measure the capacity to reduce Fe^3+^ to Fe^2+^. The berries antioxidant capacity varied significantly (*p* < 0.05) from 367.43 ± 3.09 to 93.14 ± 1.76 µg AAE/g. The highest antioxidant activity was recorded in blueberries (367.43 ± 3.09 µg AAE/g_f.w._) followed by blackberries (294.24 ± 3.20 µg AAE/g), strawberries (121.51 ± 2.10 µg AAE/g), and raspberries (93.14 ± 1.76 µg AAE/g). Previously in Lal, et al. [47] study, the antioxidant capacity of strawberries ranged between 326.06–701.13 mg AAE/100 g_fw_. The 30% ethanolic extract of blueberries and raspberries had antioxidant activity of 2.39 mg (AAE)/g and 2.32 mg (AAE)/g respectively [20]. The blackberries grown in Mexico ranged from 158.7–285.2 mol CE/g [48], which are also comparable to our study.

In the ABTS assay, the antioxidant ability is measured by reaction of the extracts with ABTS^+^ radical cation generated in the system [30]. In ABTS, the highest antioxidant ability observed in strawberries was 3.67 ± 0.14 mg AAE/g, followed by blueberries with 2.32 ± 0.09 mg AAE/g, blackberries with 1.73 ± 0.04 mg AAE/g and raspberries with 1.71 ± 0.11 mg AAE/g. In Leong and Shui [49] study, ethanolic extract of strawberries had 472 mg AAE/100 g, which is similar to the values reported in our study. The antioxidant ability of strawberries ranged between 2.25–19.58 mg AAE/g_d.w._ [45], blackberries 5422.38 mg AAE/100 g [50], blueberries 1.60 mg AAE/g [20] and 1.83 mg AAE/g_d.w._ raspberries [20]. In the previous study, blackberries showed higher value when compared to our study. These differences might be due different growing region, extraction solvent because different solvents were used to extract berries phenolics and performed antioxidant activities which might affect the extraction rate and overall antioxidant potential.

In the total antioxidant capacity (TAC) assay, the blueberries had the highest total antioxidant at 1.47 ± 0.20 mg AAE/g followed by raspberries (1.21 ± 0.01 mg AAE/g), blackberries (1.03 ± 0.09 mg AAE/g) and strawberries (0.97 ± 0.09 mg AAE/g). In a previous study led by Huang, et al. [51], the total antioxidant capacity in the methanolic extract of blueberries 14.98 mmol Trolox/100 g, blackberries 11.48 mmol Trolox/100 g and strawberries 4.44 ± 0.45 mmol Trolox/100 g_d.w._ were less than the values recorded in our study. The TAC of blackberry and blueberry was recoded as 6125.7 and 4814.6 mg AAE/100 g_d.w._, respectively by Lee, et al. [52], which is higher than the values recorded in our study. The water-soluble and insoluble TAC of strawberries were 430–900 and 390–1040 Vitamin E (TE μmol/100 g)_._, respectively, demonstrated by previous study [53].

### 3.3. LC-MS Characterization

Qualitative analysis and identification of the phenolic compounds from four different berries were carried out using LC-ESI-QTOF-MS/MS in both positive (ESI^+^) and negative (ESI^-^) ionization modes. The phenolic compounds were tentatively identified based on their *m*/*z* and MS spectra using an Agilent LC-MS mass hunter qualitative software and the Personal Compounds Database and Library (PCDL) (Supplementary data, Figures S1 and S2). The criteria for the compounds to be further analysed were the mass error < 5 ppm and PCDL library score more than 80, thereby, compounds were further identified using MS/MS identification and *m*/*z* characterization (Table 2). In the current study, total of 65 phenolic compounds were identified in 4 different berries including phenolic acids (19), flavonoids (33), other polyphenols (7), lignans (5) and stilbene (1).

#### 3.3.1. Phenolic Acids

In this study, a total of 19 phenolic acids including hydroxybenzoic acids (8), hydroxycinnamic acids (8), hydroxyphenylacetic acids (2), hydroxyphenylpropanoic acids (1) were identified and characterised in four berries.

##### Hydroxybenzoic Acids

Compound 1,2,3 and 5 were tentatively characterised as gallic acid, gallic acid 4-*O*-glucoside, 2-hydroxybenzoic acid and 2,3-dihydroxybenzoic acid respectively and the compounds were present in negative ionisation mode. The compounds have precursors ions at *m*/*z* 169.0148 (Compound 1), *m*/*z* 331.0655 (Compound 2), *m*/*z* 137.0247 (Compound 3) and *m*/*z* 153.0198 (Compound 5). Further, MS/MS analysis showed that the product ions at 125, 93 and 109 due to the loss of CO_2_ (44 Da) from precursor ions whereas product ions at 169 due to the loss of hexosyl moiety (162 Da) [54,55,56]. Gallic acid 4-*O*-glucoside was identified in strawberries and blackberries, whereas the compounds gallic acid and 2,3-dihydroxybenzoic acid were only present in strawberries, however, the compound 2-hydroxybenzoic acid was present in strawberries, raspberries and blueberries. In previous studies, gallic acid 4-*O*-glucoside presence was observed in blueberries and bilberries [57], compound gallic acid found in various maturity stages in strawberries [58] and 2,3-dihydroxybenzoic acid was observed in hops and juniper berries [21]. Compound 4 identified as protocatechuic acid 4-*O*-glucoside (*m*/*z* 315.0707) was present in both modes and the product ions at *m*/*z* 153 indicating the loss of hexosyl moiety (162 Da) from precursor molecule [54] and was only detected in strawberries. Williamson and Clifford [59] also reported the presence of protocatechuic acid 4-*O*-glucoside in blackcurrants.

##### Hydroxycinnamic Acids and Other Phenolic Acid Derivatives

In current study, the observed hydroxycinnamic acids had eight compounds with antioxidant potential. Compound 9 was identified as 1,5-dicaffeoylquinic acid ([M − H]^−^ *m*/*z* at 515.1198) observed in both modes. The product ions were at *m*/*z* 353, *m*/*z* 335, *m*/*z* 191, *m*/*z* 179 due to the loss of [M-H-C_9_H_6_O_3_], [M-H-C_9_H_8_O_4_], [M-H-C_18_H_12_O_6_] and [M-H-C_16_H_16_O_8_] from the precursor molecule, respectively [60] and had been identified in strawberries, raspberries, blueberries. 3-Feruloylquinic acid (Compound 10, precursor ([M − H]^−^ *m*/*z* at 367.1038) was present in strawberries and raspberries, confirmed by the fragments at *m*/*z* 298, *m*/*z* 288, *m*/*z* 192 and *m*/*z* 191, corresponding to the loss of [M-H-3H_2_O_2_-CH_3_], [M-H-H_2_O-CH_3_-HCOOH], [M-H-C_7_H_11_O_5_] and [M-H-C_10_H_8_O_3_], respectively [61] and previously observed in cherries [57]. 3-caffeoylquinic acid (Compound 12) with precursor [M –H]^−^ *m*/*z* at 353.0884 present in strawberries and raspberries, yielded product ions at *m*/*z* 253, *m*/*z* 190 and *m*/*z* 144 due to the corresponding loss of HCOOH-3H_2_O, C_6_H_5_O_2_-3H_2_O and C_7_H_11_O_6_-H_2_O, respectively, from the precursor molecule [61]. Compound 14 with the precursor ion at [M − H]^−^ *m*/*z* 179.0349 had been identified, and the fragment peaks at *m*/*z* 143 and *m*/*z* 133 due to the loss of 2H_2_O and HCOOH further confirmed the compound as caffeic acid and was present only in strawberries [61]. Previously, caffeic acid was found in chokeberries, raspberries and strawberries and was the major phenolic compound in saskatoon berries and wild blueberries [62]. *m*-Coumaric acid identified as compound 16 ([M − H]^−^ *m*/*z* at 163.0392), was found in all the four berries and the characteristic fragment peak was at *m*/*z* 119, corresponding to the loss of CO_2_ [63]. Jakobek, et al. [64] also observed the presence of *m*-coumaric acid in blueberries, strawberries and red raspberries.

#### 3.3.2. Flavonoids

A total of 33 flavonoids were identified including flavanols (4), flavones (2), flavanones (3), flavonols (6), dihydrochalcones (1), dihydroflavonols (1), anthocyanins (7) and isoflavonoids (9).

##### Flavanols

Three flavanols including compound 21, 22 and 23 were detected in the berries and present in both modes of ionisation. Compound 21 identified as 3′-*O*-methylcatechin with precursor [M − H]^−^ *m*/*z* at 303.0873 was only found in blueberries. The product ions at *m*/*z* 271 and *m*/*z* 163 were due to the loss of CH_3_OH and C_6_H_5_O_2_, respectively [65]. Compound 22 identified as procyanidin dimer B1 was present in strawberries, blueberries, blackberries with precursor [M − H]^−^ *m*/*z* at 577.1348 and the compound was identified upon the loss of phloroglucinol from the precursor molecule [66]. Previously, minor amounts of procyanidin dimer B1 was found in yellow raspberries [67]. Prodelphinidin dimer B3 (Compound 23, [M − H]^−^ *m*/*z* 611.1409), were identified in strawberries, blueberries and blackberries. The formation of peak at *m*/*z* 469 was due to the heterocyclic ring fission followed by removal of phloroglucinol whereas the peaks at *m*/*z* 311 and *m*/*z* 291 were due to the reduction into monomers through quinone methide fission cleavage and due to the loss of –OH group from gallocatechin respectively [68]. Li and Beta [69] reported the presence of prodelphinidin dimer B3 in whole-grain barley flour, however, to our best acknowledge, this is the first time to report the presence of this compound in berries.

##### Flavanones and Flavonols

Neoeriocitrin (Compound 26, [M − H]^−^ at *m*/*z* at 595.1674) was present in both mode and identified in strawberries, raspberries and blackberries. Based on MS/MS study, neoeriocitrin was confirmed by product ions at *m*/*z* 431 and *m*/*z* 287 due to the loss of H_2_O and glucoside [70]. Previously, the compound was identified and quantified in grapefruit juice [71].

Compound 29 with precursor at [M − H]^−^ *m*/*z* 463.0881 was identified as myricetin 3-*O*-rhamnoside and present in strawberries. The further confirmation was achieved by the fragment peak at 317 due to the loss of rhamnoside [72]. Serreli, et al. [73] also observed myricetin 3-*O*-rhamnoside in white myrtle berries. Compound 30 (Myricetin 3-*O*-galactoside with [M − H]^−^ *m*/*z* at 479.0841) present in strawberries and blueberries was identified by the product ion at *m*/*z* 317 due to the loss of glucoside [74]. Compound 32 was identified as quercetin 3-*O*-(6”-malonyl-glucoside) based on the precursor ion [M + H]^+^ at *m*/*z* 551.1038. Upon the analysis of MS/MS data, this compound was confirmed by the peak fragment at *m*/*z* 303 corresponding to the loss of malonyl-hexose unit [75]. Previously, quercetin 3-*O*-(6”-malonyl-glucoside) compound was identified in red chicory [76].

##### Dihydrochalcones, Dihydroflavonols and Anthocyanins

Phloridzin (compound 35, [M − H]^−^ *m*/*z* 435.1279) with peak fragmentation at *m*/*z* 273 due to the consecutive loss of glucoside confirms the molecule and was present in strawberries, blackberries and blueberries [77]. In previous studies, phloridzin was reported in apple flesh and peel [78] and was also confirmed in strawberries [79]. Compound 36 was identified as dihydroquercetin [M − H]^−^ *m*/*z* at 303.0508) based the fragment peaks at *m*/*z* 285 [M-H-H_2_O], *m*/*z* 275 [M-H-CO] and *m*/*z* 151 [M-H-RDA cleavage] [80]. Previously, Suh, et al. [81] found compound dihydroquercetin abundant in chokeberries and honeyberries.

Anthocyanins are mostly water-soluble phenolics and responsible for the color formation including red, blue and purple colors in different fruits and vegetables [82,83]. Anthocyanins, particularly glucosides and galactosides of cyanidin, peonidin, delphinidin, petunidin and malvidin have remarkable antioxidant potential. The antioxidant activity is high in anthocyanins when compared to other flavonoids due to their positively charged oxygen atom [84]. During ripening of different berry fruits, anthocyanins increase in production whereas the other phenolic compounds decrease including (−)-epicatechin, (+)-catechin and dimeric proanthocyanidins [85].

##### Isoflavonoids

Compound 45 present in both modes were tentatively identified as violanone present in strawberries, raspberries and blueberries with precursor at ([M − H]^−^ at *m*/*z* 315.0872). MS/MS analysis confirmed the compound 45 by the presence of the product ions at *m*/*z* 300, *m*/*z* 285 and *m*/*z* 135 corresponding to the loss of CH_3_, 2CH_3_ and C_10_H_12_O_3_ [86]. Violanone has been isolated from *Dalbergia oliveri* previously and used in traditional Thai medicine for treatment of chronic ulcer [87]. 3′-Hydroxygenistein (Compound 49, precursor ion [M + H]^+^ at *m*/*z* 287.0547) was identified by the product ions at *m*/*z* 269 and *m*/*z* 259 due to the loss of H_2_O and CO [88]. This compound was present in raspberries, blueberries and blackberries.

#### 3.3.3. Other Polyphenols

The MS/MS experiment confirmed the compound 53 as coumarin ([M + H]^+^ at *m*/*z* 147.0441) due to the product ions at *m*/*z* 103 and *m*/*z* 91 corresponding to the loss of CO_2_ and 2CO [89] present in blueberries. Javeri and Chand [90] reported that coumarin was an important component in turmeric. Compound 54 was tentatively identified as esculetin by the precursor ion [M − H]^−^ at *m*/*z* 177.0190 in strawberries. In MS/MS study, the product ions were at *m*/*z* 149 (M-H-CO), *m*/*z* 133 (M-H-CO_2_) and *m*/*z* 89 (M-H-2 CO_2_) [91]. Previously compound esculetin was reported to be present in *Vaccinium myrtillus* (bilberries) and *V. gaultherioides* (false or bog bilberries) [92]. Compound 56 (demethoxycurcumin) was only detected in the negative mode with [M − H]^−^ *m*/*z* at 337.1091 present in blueberries. In MS/MS analysis, the product ion formed at *m*/*z* 217 was corresponding to the loss of C_8_H_8_O (120 Da) from the parent ions [93].

#### 3.3.4. Lignans

Schisandrin C (Compound 61) and schisantherin A (Compound 64) were only identified in positive ionisation mode at *m*/*z* 385.1647 and *m*/*z* 537.2119. Further analysis of MS/MS confirmed the presence of schisantherin C based on product ions at *m*/*z* 370, *m*/*z* 315 and *m*/*z* 300 corresponding to the loss of CH_3_, C_5_H_10_, C_5_H_10_ [94], while product ions at *m*/*z* 519, *m*/*z* 415, *m*/*z* 385 and *m*/*z* 371 were characterized as schisantherin A due to the loss of H_2_O, C_6_H_5_COOH, C_6_H_5_COOH-CH_2_O and C_6_H_5_COOH-C_2_H_4_O. Schisandrin C and schisantherin A were only identified in blackberries. The above compounds were also identified in *Schisandra chinensis* used in Chinese medicine for years [95]. Compound 60 identified as pinoresinol was found only in blueberries with the precursor ion at *m*/*z* 357.1331 and obtained product ions at *m*/*z* 342 [M-H-CH_3_], *m*/*z* 327 [M-H-C_2_H_6_], *m*/*z* 313[M-H-CO_2_] and *m*/*z* 221 [M-H-C_8_H_8_O_2_] [60]. Compound 63 (Deoxyschisandrin) present in negative mode at *m*/*z* 415.2146 was found in strawberries. The compound was identified and confirmed by MS/MS analysis upon the loss of CH_3_, C_5_H_10_, C_5_H_10_−OCH_3_ and C_5_H_10_−OCH_3_−CH_3_ [94]. Lee, et al. [96] reported the presence of deoxyschizandrin in Schisandra berries.

### 3.4. Distribution of Phenolic Compounds in Berries

**Figure 1:**
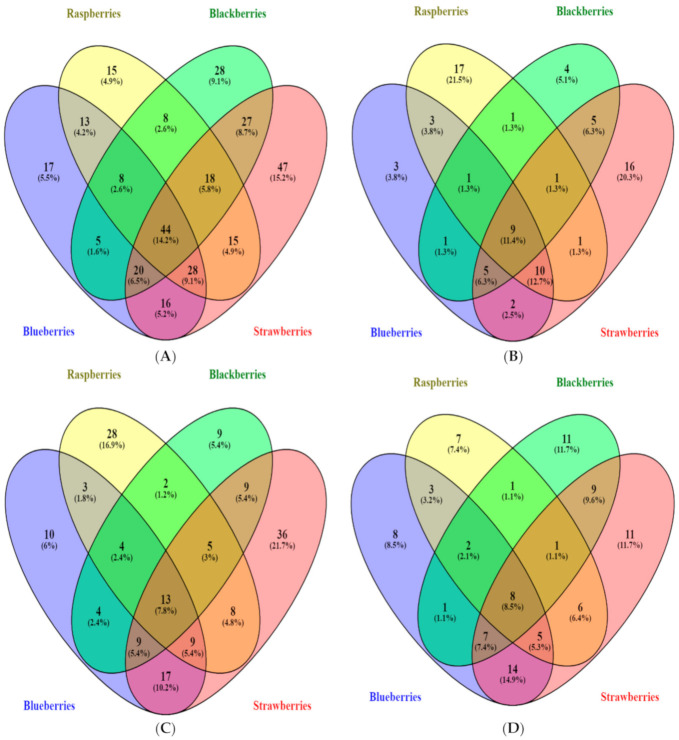
Venn diagram of phenolic compounds presented in different fruit berries. (**A**) shows the relations of total phenolic compounds present in different berries samples (**B**) shows the relations of phenolic acids among the berries. (**C**) shows the relations of flavonoids present in berry samples (**D**) shows the relations of other phenolic compounds present in different berry samples.

Berries contain a wide range of phenolic compounds in different conjugated forms, a fact that makes their simultaneous analysis a difficult task, therefore, researchers have established a keen interest in the distribution of phenolic compounds in berries. The Venn diagrams (Figure 1) were developed according to the number of phenolic compounds that had been detected in blueberries (blue), strawberries (red), raspberries (green) and blackberries (yellow).

In the Venn diagram of total phenolic compound, the unique compounds in strawberries, blackberries, blueberries, and Raspberries are 47 (15.2%), 28 (9.1%), 17 (5.5%) and 15 (4.9%) respectively. The maximum overlapping phenols were 44 (14.2%) that were shared among the four berries. The lowest number of overlapped compounds were 5 (1.6%) phenols in blackberries and blueberries followed by raspberries with blackberries and blueberries. Previous researchers, found that blackberries, raspberries and strawberries contain similar amount of total phenolic compounds [97], but another study found that blueberries had the highest polyphenol [98]. In our study, blueberries showed highest phenolic content compared to other berries. Whereas, other researchers found that the blackberries have greater concentration of total phenolic compound than strawberries and raspberries, which are grown in tropical conditions [99]. Croge, et al. [100] found fruits grown in temperate region have higher polyphenol content.

A total of 11.4% phenolic acids were common among the four berries, whereas 12.7% of phenolic acids were common among blueberries, raspberries, and strawberries. The presence of unique compounds in blueberries, strawberries, raspberries, and blackberries were 3 (3.8%), 16 (20.3%), 17 (21.5%) and 4 (5.1%), respectively. The flavonoid presented in the Venn diagram consisted of 13 flavonoids common among four berries, whereas the unique flavonoids were high in strawberries and raspberries. The highest similarity of flavonoids was among blueberries and strawberries and on the other hand the lowest common flavonoids were among blueberries and blackberries. According to the previous studies, the strawberries obtained lower content of anthocyanin as compared to blueberries, blackberries and raspberries [101]. The anthocyanin concentration accumulated was maximum at the ripening stage and around 25 anthocyanins was reported in the strawberries [102]. In our study, the total flavonoid content was present in blueberries higher than other berries.

Among the other polyphenols, 8 polyphenols were commonly found in four berries. Blueberries and strawberries had 14 polyphenols overlapped followed by 7 polyphenols common in blueberries, strawberries, and blackberries. The unique polyphenols were present in blueberries (8.5%), raspberries (7.4%), strawberries (11.7%) and blackberries (11.7%). As per our best knowledge, we did not find any related studies on the characterization of other polyphenols from fruit berries.

### 3.5. Heatmap and Hierarchical Cluster Analysis of Quantified Phenolics in Berries

**Figure 2:**
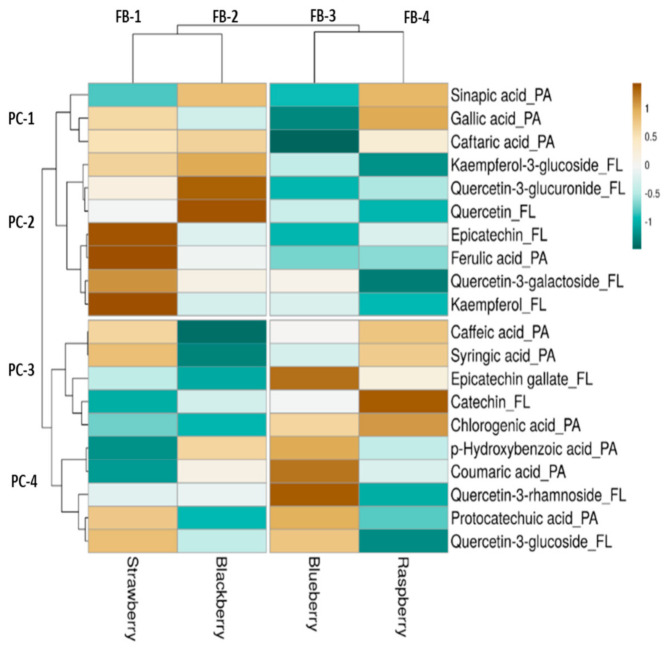
Heatmap showing phenolic compounds’ distribution and concentration among four samples of berries. Darker brown boxes mean concentrations are higher among different berries samples. Blue boxes mean lower concentrations. PA: phenolic acids; FL: flavonoids; FB 1–4: fruit berries; PC 1–4: phenolic compound clusters.

A heat map (Figure 2) was constructed along with hierarchical clusters for further analysing HPLC-PDA quantified phenolic compounds in fruit berries. Twenty phenolic compounds were quantified consisting of ten phenolic acids and ten flavonoids (Supplementary Table S1).

The hierarchically clustered heat map of the phenolic compounds of the fresh grown berries including strawberries, raspberries, blueberries and blackberries were generated. The axis of the map had samples and phenolic compounds; therefore, the pattern of branching showed the similarity among them and each branch point shows a divergence. The darker brown colour has the higher content (catechin and chlorogenic acid) and the blue colour had lower concentration; the colour difference also showed the difference among the berries.

The phenolic compounds were clustered into 4 groups of PC-1, PC-2, PC-3, and PC-4. As the branches divides and forms subgroups the similarity among the compounds increases. The phenolic acids (caffeic acid, syringic acid, chlorogenic acid) and flavonoids (epicatechin, quercetin-3-galactoside, kaempferol) showed great similarity. Whereas phenolic acids (gallic acid, syringic acid) and flavonoids (kaempferol-3-glucoside, quercetin-3-glucoside) have shown high dissimilarity. Blueberries showed higher content of phenolic acids (*p*-hydroxybenzoic acid and *p*-coumaric acid) and flavonoids (quercetin-3-rhamnoside and epicatechin) in the heat map and similar to the in vitro studies.

In phenolic acids, presence of gallic acid was high in raspberries compared to strawberries, blackberries, and blueberries. Huang, Zhang, Liu and Li [51] showed the presence of gallic acid in strawberries and blackberries, whereas, Sellappan, et al. [103] study showed that rabbit-eye blueberries had high concentration of gallic acid compared to blackberries. Blueberries had higher concentration of *p*-hydroxybenzoic acid compared to blackberry and was least in strawberries. Previous study of Huang, Zhang, Liu and Li [51] also found that the blueberries had high concentration of *p*-hydroxybenzoic acid and in our study the in vitro assays showed higher phenolic content. In our study, caffeic acid and chlorogenic acid concentration was high in raspberry but not detected in blackberry. Previous study showed that the blueberries had higher concentration of caffeic acid and chlorogenic acid [103,104] and lowest in blackberries [64]. Ferulic acid and *p*-coumaric acid concentration were high in strawberry and blueberry, respectively. Jakobek, Šeruga, Novak and Medvidović-Kosanović [64] showed the presence of low concentration of ferulic and *p*-coumaric acid in blackberries. Caftaric acid and sinapic acid were detected in blackberries, raspberries, strawberries, and negligible concentration or not detected in blueberries. Protocatechuic acid was found in all four berries, whereas syringic acid was not detected in blackberries.

In flavonoids, quercetin and its derivates including quercetin-3-glucuronide, quercetin-3-galactoside, quercetin-3-glucoside and quercetin-3-rhamnoside were identified in all four berries. Blackberries had highest concentration of quercetin and quercetin-3-glucuronide followed by strawberries. Previously, quercetin-3-rhamnoside was reported higher in blueberries [51] as compared to our results. Quercetin was detected in all four berries and previous study showed the presence in raspberries and strawberries [105]. Kaempferol presence was identified high in strawberries and its derivative kaempferol-3-glucoside was detected in blackberries. In the previous studies, the presence of kaempferol was detected in strawberries. Presence of Epicatechin and catechin was observed in all four berries [106,107].

## 4. Conclusions

In conclusion, all the selected four Australian berries have high phenolic contents and antioxidant potential. The in vitro assays (TPC, TFC, DPPH, FRAP, TAC) showed that blueberries compared to other berries had higher phenolic acids and antioxidant content. The LC-ESI-QTOF-MS/MS identified 65 phenolic compounds in the four berries. The quantification by HPLC showed the quantity of phenolic compound present and the phenolic acid (*p*-hydroxybenzoic) and flavonoid (quercetin-3-rhamnoside) were higher in blueberries. According to the results obtained, berries can have a positive benefit when used in food and nutraceutical industries. To commercialise the ingredients, further analysis can be done on bioavailability, bio accessibility and toxicology studies.

## Figures and Tables

**Table 1:** Estimation of phenolic content and antioxidant activity present in berries.

Antioxidant Assays	Blueberries	Strawberries	Blackberries	Raspberries
TPC (mg GAE/g)	2.93 ± 0.07 ^a^	1.92 ± 0.07 ^b^	1.81 ± 0.08 ^c^	1.52 ± 0.12 ^d^
TFC (µg QE/g)	70.31 ± 1.21 ^a^	14.31 ± 0.13 ^d^	30.12 ± 0.13 ^b^	22.98 ± 0.07 ^c^
TTC (mg CE/g)	7.41 ± 0.09 ^b^	2.37 ± 0.09 ^c^	11.32 ± 0.13 ^a^	0.97 ± 0.13 ^d^
DPPH (mg AAE/g)	1.69 ± 0.09 ^a^	1.11 ± 0.12 ^c^	1.12 ± 0.07 ^c^	1.41 ± 0.11 ^b^
FRAP (µg AAE/g)	367.43 ± 3.09 ^a^	121.51 ± 2.10 ^c^	294.24 ± 3.20 ^b^	93.14 ± 1.76 ^d^
ABTS (mg AAE/g)	2.32 ± 0.09 ^b^	3.67 ± 0.14 ^a^	1.73 ± 0.04 ^c^	1.71 ± 0.11 ^c^
TAC (mg AAE/g)	1.47 ± 0.20 ^a^	0.97 ± 0.09 ^d^	1.03 ± 0.09 ^c^	1.21 ± 0.01 ^b^

The data shown in the table as mean ± standard deviation (*n* = 3); Lettering (^a,b,c,d^) indicated the significant difference in the means (*p* < 0.05) using a one-way analysis of variance (ANOVA) and Tukey’s HSD test. GAE: gallic acid equivalents; QE: quercetin equivalents; CE: catechin equivalents; AAE: ascorbic acid equivalents; TPC: Total phenolic content; TFC: total flavonoid content; TTC: total tannin content; DPPH: 2,2′-diphenyl-1-picrylhydrazyl; FRAP: ferric reducing antioxidant power, ABTS: 2,2′-azinobis-(3-ethylbenzo-thiazoline-6-sulfonic acid; TAC: total antioxidant content.

**Table 2:** Characterization of phenolic compounds in different Berries by LC-ESI-QTOF-MS/MS.

No.	Proposed Compounds	Molecular Formula	RT (min)	Ionization (ESI^+^/ESI^−^)	Molecular Weight	Theoretical (*m*/*z*)	Observed (*m*/*z*)	Error (ppm)	MS^2^ Product Ions	Berries
**Phenolic acid**										
**Hydroxybenzoic acids**										
1	Gallic acid	C_7_H_6_O_5_	6.956	** [M − H]^−^	170.0215	169.0142	169.0148	3.5	125	* STRB
2	Gallic acid 4-*O*-glucoside	C_13_H_16_O_10_	10.236	[M − H]^−^	332.0743	331.0670	331.0655	−4.5	169, 125	* STRB, BLKB
3	2-Hydroxybenzoic acid	C_7_H_6_O_3_	10.932	** [M − H]^−^	138.0317	137.0244	137.0247	2.2	93	* STRB, RASB, BLUB
4	Protocatechuic acid 4-*O*-glucoside	C_13_H_16_O_9_	12.539	** [M − H]^−^	316.0794	315.0721	315.0707	−4.4	153	* STRB
5	2,3-Dihydroxybenzoic acid	C_7_H_6_O_4_	14.394	[M − H]^−^	154.0266	153.0193	153.0198	3.3	109	* STRB
6	3-*O*-Methylgallic acid	C_8_H_8_O_5_	14.529	** [M + H]^+^	184.0372	185.0445	185.0447	1.1	170, 142	* RASB, STRB, BLUB
7	3,4-*O*-Dimethylgallic acid	C_9_H_10_O_5_	38.894	** [M + H]^+^	198.0528	199.0601	199.0596	−2.5	153, 139, 125, 111	* RASB, BLUB, BLKB
8	Paeoniflorin	C_23_H_28_O_11_	58.033	** [M − H]^−^	480.1632	479.1559	479.1577	3.8	449, 357, 327	* RASB
**Hydroxycinnamic acids**										
9	1,5-Dicaffeoylquinic acid	C_25_H_24_O_12_	4.106	** [M − H]^−^	516.1268	515.1195	515.1198	0.6	353, 335, 191, 179	* STRB, RASB, BLUB
10	3-Feruloylquinic acid	C_17_H_20_O_9_	4.653	** [M − H]^−^	368.1107	367.1034	367.1038	1.1	298, 288, 192, 191	* STRB, RASB
11	Ferulic acid	C_10_H_10_O_4_	4.821	** [M − H]^−^	194.0579	193.0506	193.0511	2.6	178, 149, 134	* RASB
12	3-Caffeoylquinic acid	C_16_H_18_O_9_	4.852	** [M − H]^−^	354.0951	353.0878	353.0884	1.7	253, 190, 144	* STRB, RASB
13	Ferulic acid 4-*O*-glucuronide	C_16_H_18_O_10_	23.672	** [M − H]^−^	370.0900	369.0827	369.0810	−4.6	193	* STRB, BLUB
14	Caffeic acid	C_9_H_8_O_4_	28.724	** [M − H]^−^	180.0423	179.0350	179.0349	−0.6	143, 133	* STRB
15	1,2,2′-Triferuloylgentiobiose	C_42_H_46_O_20_	31.127	** [M − H]^−^	870.2582	869.2509	869.2506	−0.3	693, 517	* STRB
16	*m*-Coumaric acid	C_9_H_8_O_3_	35.682	** [M − H]^−^	164.0473	163.0400	163.0392	−4.9	119	* STRB, RASB, BLUB, BLKB
**Hydroxyphenylacetic acids**										
17	3,4-Dihydroxyphenylacetic acid	C_8_H_8_O_4_	13.450	** [M − H]^−^	168.0423	167.0350	167.0344	−1.8	149, 123	* STRB
18	2-Hydroxy-2-phenylacetic acid	C_8_H_8_O_3_	40.106	** [M − H]^−^	152.0473	151.0400	151.0394	−4.0	136, 92	* STRB
**Hydroxyphenylpropanoic acids**										
19	Dihydrocaffeic acid 3-*O*-glucuronide	C_15_H_18_O_10_	12.340	[M − H]^−^	358.0900	357.0827	357.0818	−2.5	181	* STRB
**Flavonoids**										
**Flavanols**										
20	(-)-Epigallocatechin	C_15_H_14_O_7_	4.804	** [M − H]^−^	306.0740	305.0667	305.0679	3.9	261, 219	* RASB, STRB
21	3′-*O*-Methylcatechin	C_16_H_16_O_6_	11.736	** [M − H]^−^	304.0947	303.0874	303.0873	−0.3	271, 163	* BLUB
22	Procyanidin dimer B1	C_30_H_26_O_12_	19.047	** [M − H]^−^	578.1424	577.1351	577.1324	−4.7	451	* STRB, RASB, BLUB
23	Prodelphinidin dimer B3	C_30_H_26_O_14_	43.974	** [M + H]^+^	610.1323	611.1396	611.1409	2.1	469, 311, 291	* STRB, BLUB, BLKB
**Flavones**										
24	Apigenin 7-*O* apiosylglucoside	C_26_H_28_O_14_	32.285	** [M + H]^+^	564.1479	565.1552	565.1528	−4.2	296	* RASB
25	Chrysoeriol 7-*O*-glucoside	C_22_H_22_O_11_	35.368	** [M + H]^+^	462.1162	463.1235	463.1228	−1.5	445, 427, 409, 381	* BLKB
**Flavanones**										
26	Neoeriocitrin	C_27_H_32_O_15_	13.168	** [M − H]^−^	596.1741	595.1668	595.1674	1.0	431, 287	* STRB, RASB, BLKB
27	Narirutin	C_27_H_32_O_14_	38.326	** [M − H]^−^	580.1792	579.1719	579.1707	−2.1	271	* STRB
28	Hesperidin	C_28_H_34_O_15_	44.090	[M + H]^+^	610.1898	611.1971	611.1981	1.6	593, 465, 449, 303	* STRB, BLUB, RASB
**Flavonols**										
29	Myricetin 3-*O*-rhamnoside	C_21_H_20_O_12_	11.810	** [M − H]^−^	464.0955	463.0882	463.0893	2.4	317	* STRB
30	Myricetin 3-*O*-galactoside	C_21_H_20_O_13_	12.754	[M − H]^−^	480.0904	479.0831	479.0841	2.1	317	* STRB, BLUB
31	Kaempferol 3-*O*-(2′′-rhamnosyl-galactoside) 7-*O*-rhamnoside	C_33_H_40_O_19_	21.217	** [M − H]^−^	740.2164	739.2091	739.2067	−3.2	593, 447, 285	* STRB, RASB, BLUB
32	Quercetin 3-*O*-(6′′-malonyl-glucoside)	C_24_H_22_O_15_	25.423	[M + H]^+^	550.0959	551.1032	551.1038	1.1	303	* STRB
33	Quercetin-3-*O*-xylosyl-glucuronide	C_26_H_26_O_17_	43.990	** [M + H]^+^	610.1170	611.1243	611.1222	−3.4	479, 303, 285, 239	* STRB, RASB
34	Kaempferol 7-O-glucoside	C_21_H_19_O_11_	86.415	** [M − H]−	447.0927	446.0854	446.0835	−4.3	357,327,297,285	* BLKB, BLUB
**Dihydrochalcones**										
35	Phloridzin	C_21_H_24_O_10_	49.400	** [M − H]^−^	436.1369	435.1296	435.1279	−3.9	273	* STRB, BLUB, BLKB
**Dihydroflavonols**										
36	Dihydroquercetin	C_15_H_12_O_7_	12.382	** [M − H]^−^	304.0583	303.0510	303.0508	−0.7	285, 275, 151	* BLUB, STRB, RASB, BLKB
**Anthocyanins**										
37	Delphinidin 3-*O*-glucoside	C_21_H_21_O_12_	22.960	** [M + H]^+^	465.1033	466.1106	466.1117	2.4	303	* RASB
38	Cyanidin 3,5-*O*-diglucoside	C_27_H_31_O_16_	26.207	** [M + H]^+^	611.1612	612.1685	612.1700	2.5	449, 287	* STRB, RASB, BLKB
39	Peonidin 3-*O*-sambubioside-5-*O*-glucoside	C_33_H_41_O_20_	27.813	** [M + H]^+^	757.2191	758.2264	758.2245	−2.5	595, 449, 287	* RASB, BLKB
40	4-*O*-Methyldelphinidin-3-*O*-D-glucoside	C_22_H_23_O_12_	29.100	[M + H]^+^	479.1190	480.1263	480.1248	−3.1	317, 303, 285, 271	* STRB
41	Isopeonidin 3-*O*-arabinoside	C_21_H_21_O_10_	32.685	[M + H]^+^	433.1135	434.1208	434.1196	−2.8	271, 253, 243	* BLUB, BLKB
42	Pelargonidin 3-*O*-rutinoside	C_27_H_31_O_14_	34.025	[M + H]^+^	579.1714	580.1787	580.1794	1.2	271, 433	* BLUB, STRB, RASB
43	Cyanidin 3-*O*-(6′′-*p*-coumaroyl-glucoside)	C_30_H_27_O_13_	50.086	** [M + H]^+^	595.1452	596.1525	596.1519	−1.0	287	* STRB, RASB, BLUB, BLKB
**Isoflavonoids**										
44	2-Dehydro-*O*-desmethylangolensin	C_15_H_12_O_4_	4.554	[M − H]^−^	256.0736	255.0663	255.0657	−2.4	135, 119	* STRB, BLUB
45	Violanone	C_17_H_16_O_6_	12.572	** [M − H]^−^	316.0947	315.0874	315.0872	−0.6	300, 285, 135	* STRB, RASB, BLUB
46	3′-O-Methylviolanone	C_18_H_18_O_6_	13.301	[M − H]^−^	330.1103	329.103	329.1033	0.9	314,299,284,256	* STRB, BLUB
47	Equol	C_15_H_14_O_3_	14.132	[M + H]^+^	242.0943	243.1016	243.1015	−0.4	255, 211, 197	* RASB
48	6-*O*-Malonylgenistin	C_24_H_22_O_13_	29.000	[M + H]^+^	518.1060	519.1133	519.1112	−4.0	271	* STRB
49	3′-Hydroxygenistein	C_15_H_10_O_6_	29.470	** [M + H]^+^	286.0477	287.0550	287.0547	−1.0	269, 259	* RASB, BLUB, BLKB
51	3′-Hydroxydaidzein	C_15_H_10_O_5_	32.205	[M + H]^+^	270.0528	271.0601	271.0604	1.1	253, 241, 225	* BLUB
50	6′′-*O*-Malonylglycitin	C_25_H_24_O_13_	41.082	** [M + H]^+^	532.1217	533.1290	533.1277	−2.4	285, 270, 253	* BLKB, BLUB
52	5,6,7,3′,4′-Pentahydroxyisoflavone	C_15_H_10_O_7_	44.007	** [M + H]^+^	302.0427	303.0500	303.0502	0.7	285, 257	* STRB, RASB, BLKB
**Other polyphenols** **Hydroxycoumarins**										
53	Coumarin	C_9_H_6_O_2_	26.474	** [M + H]^+^	146.0368	147.0441	147.0440	−0.7	103, 91	* BLUB
54	Esculetin	C_9_H_6_O_4_	27.267	[M − H]^−^	178.0266	177.0193	177.019	−1.7	149, 133, 89	* STRB
**Hydroxybenzaldehydes**										
55	*p*-Anisaldehyde	C_8_H_8_O_2_	13.850	** [M + H]^+^	136.0524	137.0597	137.0600	2.2	122, 109	* STRB, RASB
**Curcuminoids**										
56	Demethoxycurcumin	C_20_H_18_O_5_	20.648	[M − H]^−^	338.1154	337.1081	337.1091	3.0	217	* BLUB
57	Bisdemethoxycurcumin	C_19_H_16_O_4_	33.646	[M + H]^+^	308.1049	309.1122	309.1123	0.3	291, 263	* BLKB
**Other polyphenols**										
58	Arbutin	C_12_H_16_O_7_	4.148	** [M − H]^−^	272.0896	271.0823	271.0824	0.4	109	* BLUB, RASB
59	Lithospermic acid	C_27_H_22_O_12_	76.972	[M − H]^−^	538.1111	537.1038	537.1035	−0.6	493, 339, 295	* BLKB
**Lignans**										
60	Pinoresinol	C_20_H_22_O_6_	11.189	** [M − H]^−^	358.1416	357.1343	357.1331	−3.4	342, 327, 313, 221	* BLUB
61	Schisandrin C	C_22_H_24_O_6_	11.666	[M + H]^+^	384.1573	385.1646	385.1647	0.3	370, 315, 300	* BLKB
62	Sesamin	C_20_H_18_O_6_	14.676	[M − H]^−^	354.1103	353.103	353.1038	2.3	338, 163	* STRB, RASB
63	Deoxyschisandrin	C_24_H_32_O_6_	23.125	[M − H]^−^	416.2199	415.2126	415.2146	4.8	402, 347, 361, 301	* STRB
64	Schisantherin A	C_30_H_32_O_9_	81.398	[M + H]^+^	536.2046	537.2119	537.2119	0.0	519, 415, 385, 371	* BLKB
Stilbenes										
65	4-Hydroxy-3,5,4′-trimethoxystilbene	C_17_H_18_O_4_	44.923	[M + H]^+^	286.1205	287.1278	287.1287	3.1	271, 241, 225	* RASB, BLKB

* Data presented in the table are from the sample indicated with an asterisk; ** Compounds were detected in both negative [M − H]^−^ and positive [M + H]^+^ mode of ionization while only single mode data was presented. Berry samples mentioned in abbreviations are Strawberry “STRB”, Raspberry “RASB”, Blueberry “BLUB’’ and Blackberry “BLKB”.

## Supplementary Materials

The following are available online at https://www.mdpi.com/2076-3921/10/1/26/s1, Figure S1: LC-ESI-QTOF-MS/MS basic peak chromatograph (BPC) for characterization of phenolic compounds of berries. Figure S2. Extracted ion chromatogram of strawberries and their mass spectrum. Table S1: Quantification of targeted phenolics in berries through HPLC-PDA.
