# Supplementary material for: Screening of Phenolic Compounds in Australian Grown Berries by LC-ESI-QTOF-MS/MS and Determination of Their Antioxidant Potential
Source: Antioxidants (Basel). 2020 Dec 29;10(1):26. doi: 10.3390/antiox10010026 (PMC7824486; doi:10.3390/antiox10010026)
Supplement: Supplementary file 1 [file antioxidants-10-00026-s001.pdf]

antioxidants

MDPI

Supplementary data

Screening of phenolic compounds in Australian grown berries by LC-ESI-QTOF-MS/MS and determination of their antioxidant potential

Vigasini Subbiah[1], Biming Zhong[1], Malik A. Nawaz[2], Colin J. Barrow[3], Frank R. Dunshea[1,4] and Hafiz A.R. Suleria[1,3*]

[1] School of Agriculture and Food, Faculty of Veterinary and Agricultural Sciences, The University of Melbourne, Parkville 3010, VIC, Australia; vsubbiah@student.edu.au (V.S.); bimingz@student.unimelb.edu.au (B.Z); fdunshea@unimelb.edu.au (F.R.D)

[2] Commonwealth Scientific and Industrial Research Organisation (CSIRO), Agriculture and Food, 671 Sneydes Road, Private Bag 16, Werribee, Victoria, 3030, Australia; malik.nawaz@csiro.au (M.A.N)

[3] Centre for Chemistry and Biotechnology, School of Life and Environmental Sciences, Deakin University, Waurn Ponds, VIC 3217, Australia; colin.barrow@deakin.edu.au (C.J.B)

[4]Faculty of Biological Sciences, The University of Leeds, Leeds LS2 9JT, UK

* Correspondence: hafiz.suleria@unimelb.edu.au; Tel.: +61-470-439-670

Received: date; Accepted: date; Published: date

Abstract: Berries are grown worldwide with the most consumed berries being blackberries (*Rubus* spp.), blueberries (*Vaccinium corymbosum*), red raspberries (*Rubus idaeus*) and strawberries (*Fragaria* spp.). Berries are either consumed fresh, frozen, or processed into wines, juices, and jams. In recent times, researchers have focused their attention on berries due to their abundance in phenolic compounds. The current study aimed to evaluate the phenolic content and their antioxidant potential followed by characterization and quantification using LC-ESI-QTOF-MS/MS and HPLC-PDA. Blueberries were highest in TPC (2.93 ± 0.07 mg GAE/$g_{f.w}$) and TFC (70.31 ± 1.21 µg QE/$g_{f.w}$), whereas, the blackberries had the highest content in TTC (11.32 ± 0.13 mg CE/$g_{f.w}$). Blueberries had the highest radical scavenging capacities for the DPPH (1.69 ± 0.09 mg AAE/$g_{f.w}$), FRAP (367.43 ± 3.09 µg AAE/$g_{f.w}$), TAC (1.47 ± 0.20 mg AAE/$g_{f.w}$) and ABTS was highest in strawberries (3.67 ± 0.14 mg AAE/$g_{f.w}$). LC-ESI-QTOF-MS/MS study identified a total of 65 compounds including 42 compounds in strawberries, 30 compounds in raspberries, 28 compounds in blueberries and 21 compounds in blackberries. The HPLC-PDA quantification observed phenolic acid (*p*-hydroxybenzoic) and flavonoid (quercetin-3-rhamnoside) higher in blueberries compared to other berries. Our study showed the presence of phenolic acids and provides information to be utilized as an ingredient in food, pharmaceutical and nutraceutical industries.

Keywords: Fruit berries; blackberries; blueberries; red raspberries; strawberries; polyphenols; antioxidant activity; HPLC-PDA; LC-MS/MS.

(a)

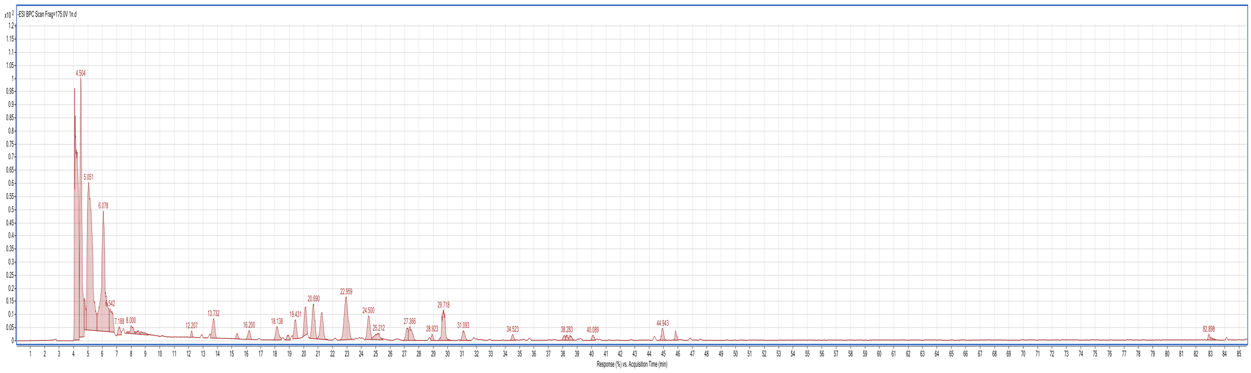

(b)

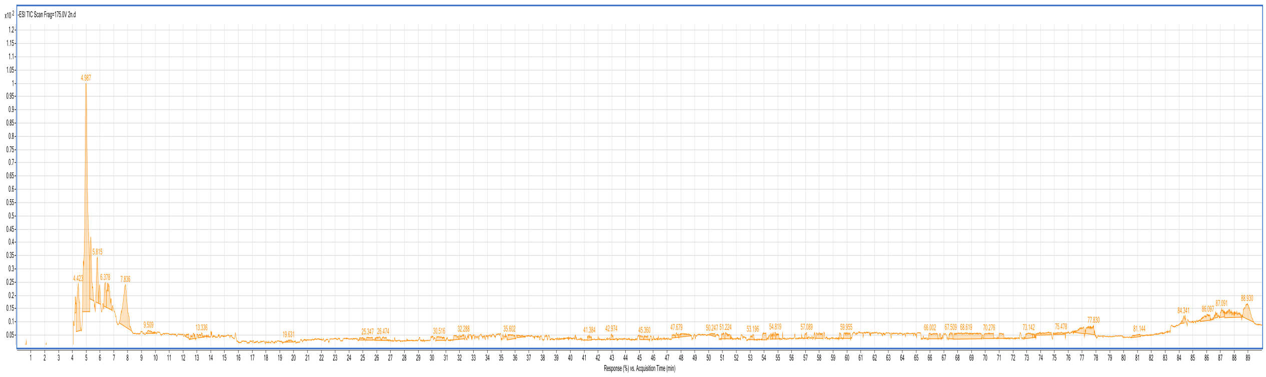

(c)

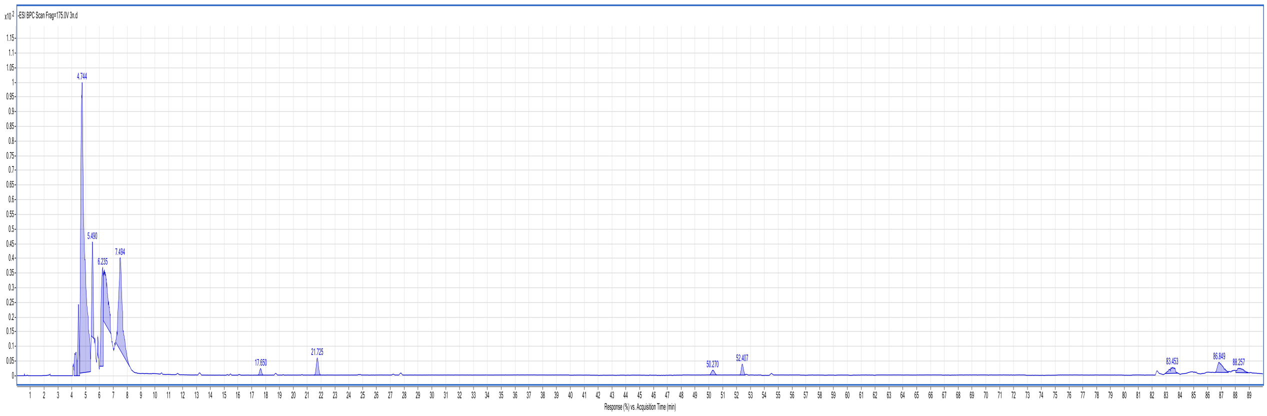

(d)

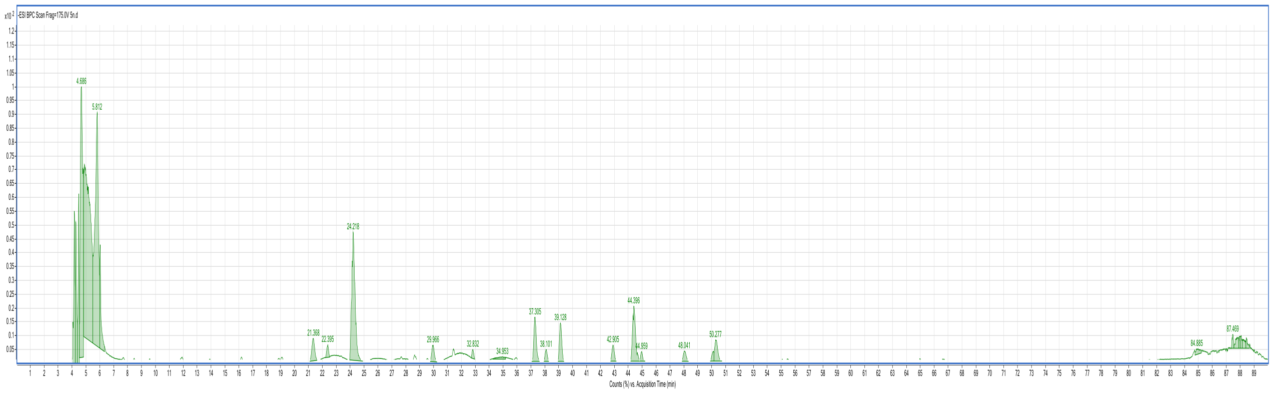

(e)

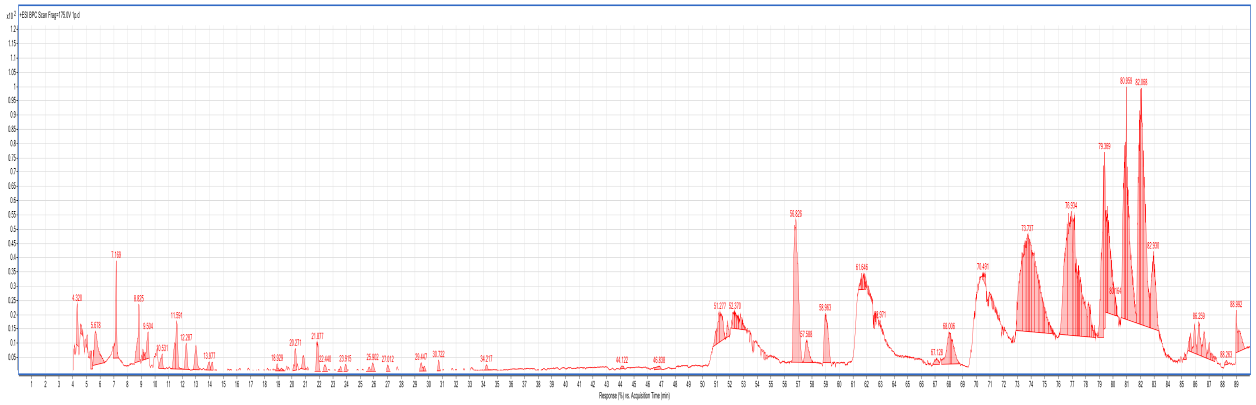
Response (%) vs. Acquisition Time (min)

(f)

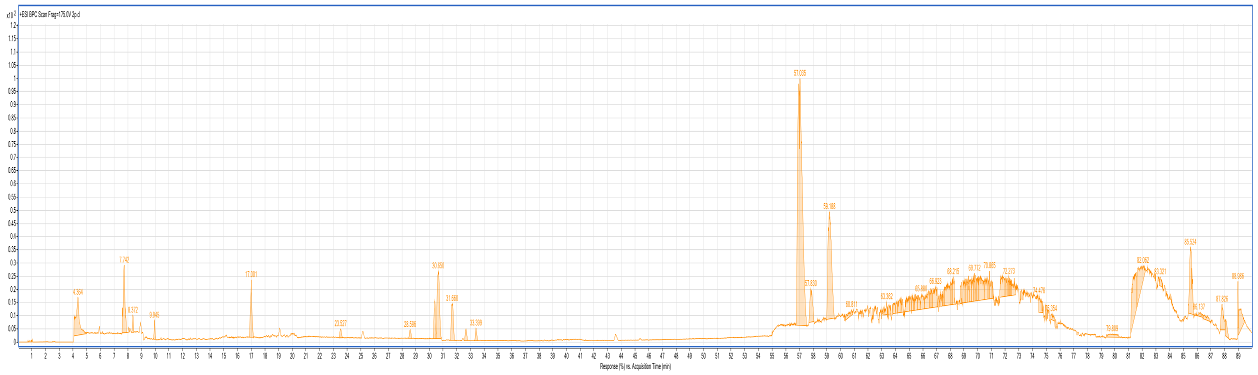
Response (%) vs. Acquisition Time (min)

(g)

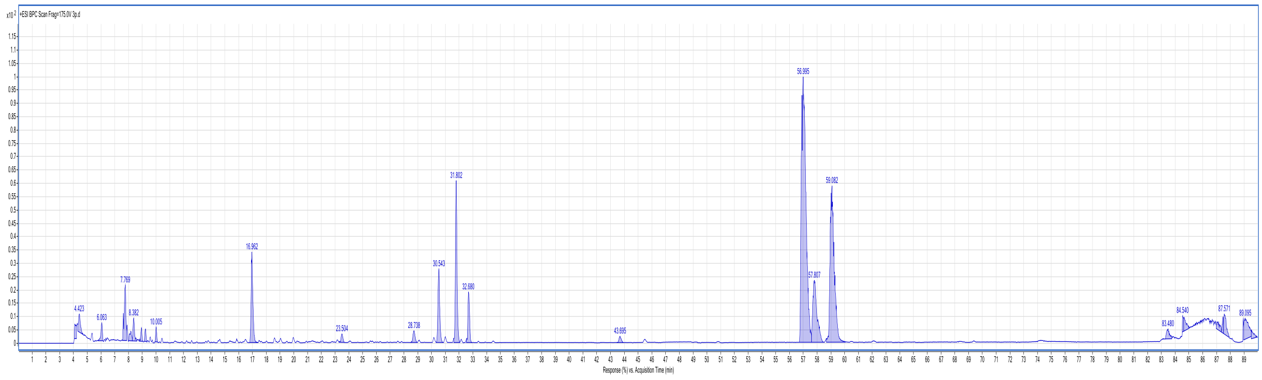
Response (%) vs. Acquisition Time (min)

(h)

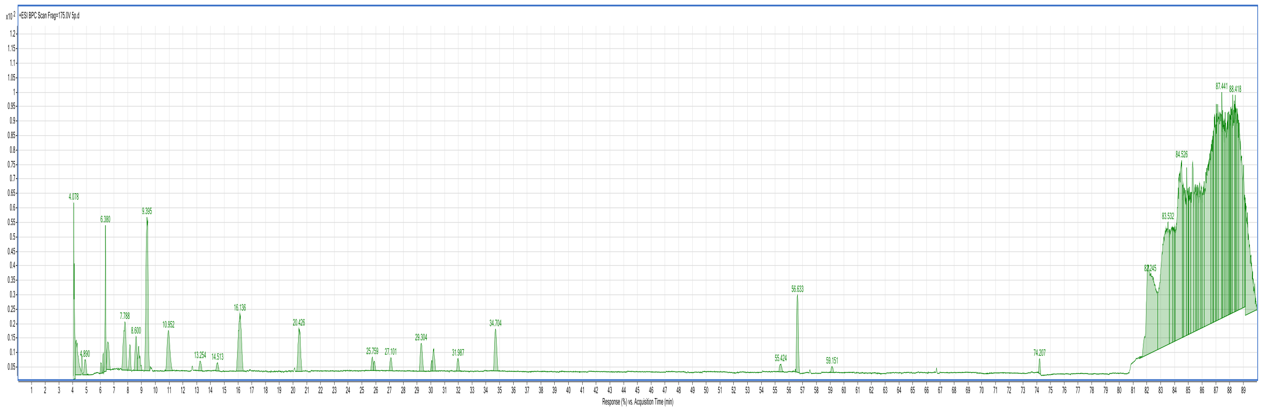
Response (%) vs. Acquisition Time (min)

Figure (1S): LC-ESI-QTOF-MS/MS basic peak chromatograph (BPC) for characterization of phenolic compounds of berries; **(a)** Strawberries in negative ionization mode; **(b)** Raspberries in negative ionization mode; **(c)** Blueberries in negative ionization mode; **(d)** Blackberries in negative ionization mode; **(e)** Strawberries in positive ionization mode; **(f)** Raspberries in positive ionization mode; **(g)** Blueberries in positive ionization mode; **(h)** Blackberries in positive ionization mode.

(a)

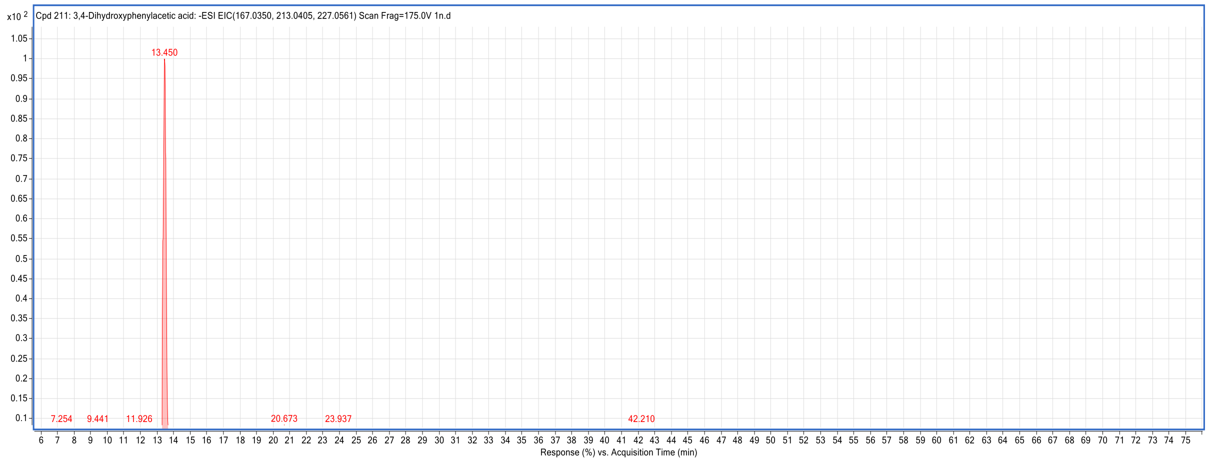

(b)

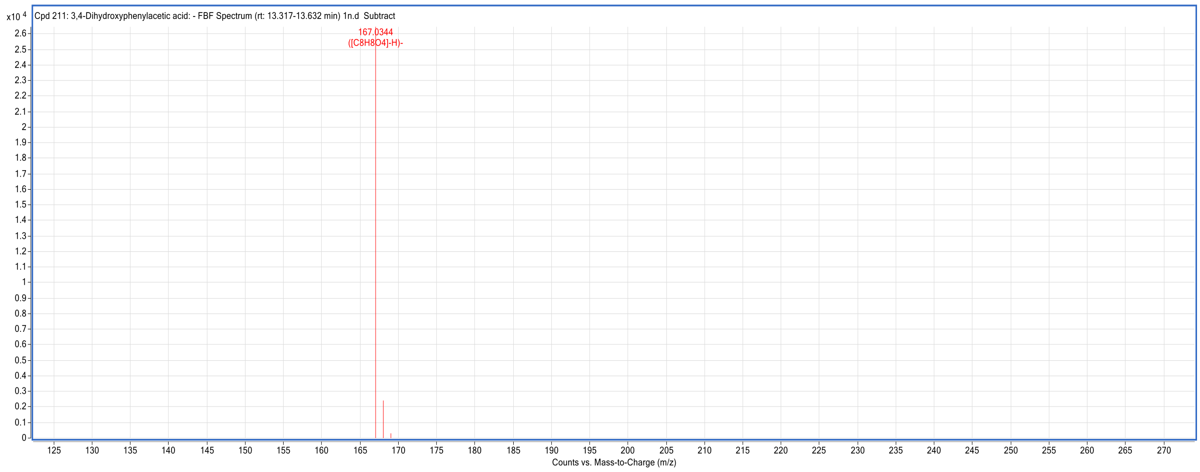

Figure (S2). Extracted ion chromatogram and their mass spectrum. (a) A chromatograph of 3,4-Dihydroxyphenylacetic acid (Compound 17, Table 2), Retention time (RT = 13.450 min) in the negative mode of ionization ($ESI^-/[M-H]^-$) identified and characterized in strawberries; **(b)** Mass spectra of 3,4-Dihydroxyphenylacetic acid showing an observed *m/z* 167.0344 in strawberries.

Table (S1): Quantification of targeted phenolics in berries through HPLC-PDA.

No.	Compound Name	Molecular Formula	Strawberry (mg/100g f.w)	Blueberry (mg/100g f.w)	Raspberry (mg/100g f.w)	Blackberry (mg/100g f.w)	Polyphenol Classes
1	Gallic acid	$C_7H_6O_5$	33.08 ± 1.65[b]	5.68 ± 0.34[d]	38.42 ± 2.69[a]	19.27 ± 1.54[c]	Phenolic acid
2	Protocatechuic acid	$C_7H_6O_4$	38.42 ± 2.31[b]	41.45 ± 2.07[a]	16.71 ± 1.00[c]	14.56 ± 1.02[d]	Phenolic acid
3	Caftaric acid	$C_{13}H_{12}O_9$	10.10 ± 0.81[a]	-	8.88 ± 0.44[b]	10.88 ± 0.65[a]	Phenolic acid
4	*p*-Hydroxybenzoic acid	$C_7H_6O_3$	20.98 ± 1.47[d]	59.89 ± 4.79[a]	35.09 ± 2.11[c]	53.78 ± 2.69[b]	Phenolic acid
5	Chlorogenic acid	$C_{16}H_{18}O_9$	6.75 ± 0.34[c]	13.52 ± 0.81[b]	15.63 ± 1.09[a]	5.53 ± 0.44[d]	Phenolic acid
6	Caffeic acid	$C_9H_8O_4$	9.95 ± 0.60[b]	6.75 ± 0.34[c]	10.60 ± 0.64[a]	-	Phenolic acid
7	Syringic acid	C9H10O5	20.70 ± 1.66[a]	9.95 ± 0.60[c]	19.65 ± 0.98[b]	-	Phenolic acid
8	*p*-Coumaric acid	$C_9H_8O_3$	6.01 ± 0.36[d]	25.38 ± 1.78[a]	13.33 ± 1.07[c]	16.44 ± 0.99[b]	Phenolic acid
9	Ferulic acid	$C_{10}H_{10}O_4$	4.05 ± 0.20[a]	0.97 ± 0.06[c]	1.07 ± 0.07[b]	1.89 ± 0.15[b]	Phenolic acid
10	Sinapic acid	$C_{11}H_{12}O_5$	1.11 ± 0.09[a]	0.11 ± 0.01[a]	19.65 ± 0.98[a]	18.81 ± 1.13[b]	Phenolic acid
11	Catechin	$C_{15}H_{14}O_6$	13.52 ± 0.81[d]	20.98 ± 1.47[b]	31.52 ± 2.52[a]	19.05 ± 1.14[c]	Flavonoids
12	Epicatechin	$C_{15}H_{14}O_6$	71.34 ± 5.23[a]	20.70 ± 1.66[d]	36.56 ± 2.19[c]	37.10 ± 1.85[b]	Flavonoids
13	Epicatechin gallate	$C_{22}H_{18}O_{10}$	5.48 ± 0.33[c]	19.27 ± 0.96[a]	10.60 ± 0.64[b]	0.48 ± 0.03[d]	Flavonoids
14	Quercetin-3-glucuronide	$C_{21}H_{18}O_{13}$	35.09 ± 2.46[b]	0.06 ± 0.05[d]	13.33 ± 0.80[c]	71.42 ± 3.57[a]	Flavonoids
15	Quercetin-3-galactoside	$C_{21}H_{20}O_{12}$	31.52 ± 1.89[a]	18.81 ± 1.32[c]	1.07 ± 0.08[d]	19.27 ± 1.16[b]	Flavonoids
16	Quercetin-3-glucoside	$C_{21}H_{20}O_{12}$	35.40 ± 1.77[a]	34.64 ± 2.08[b]	0.99 ± 0.07[d]	14.56 ± 1.16[c]	Flavonoids
17	Quercetin-3-rhamnoside	$C_{21}H_{20}O_{11}$	10.31 ± 0.62[b]	26.00 ± 1.30[a]	1.89 ± 0.11[c]	10.88 ± 0.76[b]	Flavonoids
18	Kaempferol-3-glucoside	$C_{21}H_{20}O_{11}$	13.38 ± 1.07[b]	6.01 ± 0.36[c]	0.48 ± 0.02[d]	15.63 ± 0.94[a]	Flavonoids
19	Quercetin	$C_{15}H_{10}O_7$	15.21 ± 1.06[b]	10.60 ± 0.85[c]	1.34 ± 0.08[d]	36.56 ± 1.83[a]	Flavonoids
20	Kaempferol	$C_{15}H_{10}O_6$	66.78 ± 4.32[a]	19.65 ± 1.37[b]	0.06 ± 0.05[c]	19.05 ± 1.14[b]	Flavonoids

All data are the mean ± SD of three replicates. Means followed by different letters ([a, b, c, d, e]) within the same column are significantly different ($p < 0.05$) of each other. Data of fruit berries were reported on a fresh weight basis (mg/100 g).
